# Increased deep sleep in a medication-free, detoxified female offender with schizophrenia, alcoholism and a history of attempted homicide: Case report

**DOI:** 10.1186/1471-244X-4-35

**Published:** 2004-10-26

**Authors:** Nina Lindberg, Pekka Tani, Pirjo Takala, Eila Sailas, Hanna Putkonen, Markku Eronen, Matti Virkkunen

**Affiliations:** 1 Institute of Biomedicine, Department of Physiology, University of Helsinki, Helsinki, Finland; 2 Institute of Clinical Medicine, Department of Psychiatry, University of Helsinki, Finland; 3 Vanha Vaasa Hospital, Vaasa, Finland

## Abstract

**Background:**

Psychiatric sleep research has attempted to identify diagnostically sensitive and specific sleep patterns associated with particular disorders. Both schizophrenia and alcoholism are typically characterized by a severe sleep disturbance associated with decreased amounts of slow wave sleep, the physiologically significant, refreshing part of the sleep. Antisocial behaviour with severe aggression, on the contrary, has been reported to associate with increased deep sleep reflecting either specific brain pathology or a delay in the normal development of sleep patterns. The authors are not aware of previous sleep studies in patients with both schizophrenia and antisocial personality disorder.

**Case presentation:**

The aim of the present case-study was to characterize the sleep architecture of a violent, medication-free and detoxified female offender with schizophrenia, alcoholism and features of antisocial personality disorder using polysomnography. The controls consisted of three healthy, age-matched women with no history of physical violence. The offender's sleep architecture was otherwise very typical for patients with schizophrenia and/or alcoholism, but an extremely high amount of deep sleep was observed in her sleep recording.

**Conclusions:**

The finding strengthens the view that severe aggression is related to an abnormal sleep pattern with increased deep sleep. The authors were able to observe this phenomenon in an antisocially behaving, violent female offender with schizophrenia and alcohol dependence, the latter disorders previously reported to be associated with low levels of slow wave sleep. New studies are, however, needed to confirm and explain this preliminary finding.

## Background

Female violent behaviour has been studied less than that of men. This is partly because women commit fewer crimes than men [1], but also because female aggression is traditionally carried out in private and domestic settings [2]. Even though the rate of violent crimes among women appears to be increasing [3,4], female homicide can still be seen as a rare phenomenon. A psychotic disorder has been observed in ca 30 % of homicidal women [5], and schizophrenia has been reported to increase the risk for homicidal violence [6]. Alcohol is often present in severe violent crimes [7] and the risk for homicidal behaviour is extremely high in women with both schizophrenia and alcohol dependency [8]. Schizophrenia patients with antisocial personality disorder represent a special high-risk subgroup that is vulnerable to severe substance abuse, psychiatric impairment and aggression as well as to legal problems [9].

Human sleep consists of two main components: rapid eye movement (REM) sleep, and non-REM sleep, the latter divided into stages 1–4 (S1–S4). Stage 3 sleep (S3) and stage 4 sleep (S4) in non-REM sleep are defined as slow-wave sleep (SWS), also called delta sleep or deep sleep. In normal sleep, REM and non-REM sleep periods alternate cyclically. Though the exact functions of the different sleep stages are unknown, it is generally accepted that SWS is the physiologically significant, refreshing part of sleep. Feelings of unwellness, either somatic or psychiatric, are frequently associated with decreased SWS.

Serious sleep disturbances are associated both with schizophrenia and alcoholism. Typical findings in both disorders are long sleep latency, reduced sleep length, long periods of waking after sleep onset, abnormalities in REM parameters as well as decreased SWS [10-12]. The reduction in SWS tends to persist after the clinical remission of psychotic symptoms [13], and it has been suggested that reduced SWS is the prevailing alteration in the sleep of patients with schizophrenia [14], although Hoffmann et al. [15] challenged this view in their recent study.

The aim of the present study was to characterize the sleep architecture in a severely violent, medication-free, detoxified woman with schizophrenia, alcoholism and a history of attempted homicide. She is the first subject in a larger study concerning sleep structure in women with this type of comorbidity.

## Case presentation

### Participants

#### The patient

The patient was a 23-year-old woman charged with attempted manslaughter. She was ordered by the court to undergo a pre-trial forensic psychiatric examination which took place in a maximum security state mental hospital. The trial records and all available background information were reviewed. Both of her parents were alcoholics and she was taken to protective custody under the age of one year. Her grandmother suffered from schizophrenia and her mother had been in a psychiatric hospital after a suicide attempt. The patient used to shoplift before the age of 15 and later she stole money from time to time. She also worked as a prostitute to earn money. She started to abuse alcohol at the age of 15. She had several boyfriends but has never married and has no children. Her personality was noticed to change before the age of 17. She started to have obsessive-compulsive behaviour, paranoid thoughts as well as depressive symptoms. She tried to kill herself by hanging and by several drug intoxications. She brutally killed her own pet. She was never hospitalized before the forensic mental examination but she irregularly visited an outpatient clinic. Despite psychiatric treatment, she impulsively tried to kill her male friend with whom she was drinking. During the psychiatric examination the diagnoses – schizophrenia paranoid type, alcohol dependence and features of antisocial personality disorder – were made by a senior forensic psychiatrist using the structured clinical interview for DSM-IV, SCID I and II [16,17]. Neither waking EEG nor brain MRI (1.5T) disclosed any abnormality. She had no somatic disorders. She had finished high school and vocational school, and was within average intelligence (WAIS-IQ total 109). She was not sentenced, but was ordered by the Finnish National Board of Medico-Legal Affairs to stay in the state mental hospital as a criminally insane patient. The sleep recordings were performed during the psychiatric examination period and the patient was completely medication-free and had abstained from alcohol and drugs for six months.

#### The controls

The control group consisted of three 23-year-old female students without criminal records or a history of physical violence. They were healthy with no signs of somatic, psychiatric, or neurological disorders. As part of a psychiatric interview, the SCID-non-patient version [18] was filled in. To exclude general diseases that could affect sleep, blood tests (including serum prolactin, thyroid function, kidney and liver function) and electrocardiograms were taken and they were within normal range, both in the patient and in controls. No history of alcohol abuse or dependence was detected in controls. The controls were asked to avoid alcohol, drugs or medication two weeks prior to the sleep examinations. Caffeine and nicotine consumption was neither restricted nor recorded.

Written consent was obtained from all participants after the study procedure had been fully explained to them. The study was approved by the local human ethics committee.

### Sleep examination

#### Polysomnography

Polysomnography (PSG) was recorded over two consecutive nights but only the second night was considered for the study. The study patient slept in a single room within the department, while controls slept in the hospital guest room. All participants were allowed to sleep as long as they wanted to. Recordings took place on an ambulatory basis; the participant had a portable recording device (Embla, Flaga hf, Reykjavik, Iceland) that was connected to the recording electrodes. The recordings were performed using the standard Rechtschaffen-Kales method [19]. The high-pass filter was 0.5 Hz and the low-pass filter 45 Hz, with a sampling rate of 100 Hz. Commercial software (Somnologica, version 2.0, Flaga hf, Reykjavik, Iceland) was used for scoring and calculation of sleep parameters. Sleep onset was defined as the first occurrence of three consecutive epochs (90 sec) of stage 1 (S1) or other sleep stages. The following parameters were calculated: time in bed, sleep latency, sleep period (time in bed – sleep latency), wake after sleep onset, total sleep time (sleep period – wake after sleep onset), sleep efficiency (total sleep time/sleep period), number of awakenings, REM latency and percentage amounts of different sleep stages (S1–S4 %, REM %). All data for the analysis were scored by the same scorer (NL), not blinded to the patient group.

#### Sleep diary

A sleep diary was used for one week during the study period to ensure a normal sleep-wake rhythm and to exclude the effects of daytime naps. The participant filled in the time of retiring to bed, estimated time of falling asleep and time of awakening in the morning for each consecutive night as well as daytime naps.

## Results and discussion

The patient's PSG recording with reduced sleep length, long sleep latency, and a high number of awakenings after sleep onset as well as reduced sleep efficiency (Table 1) is very typical for patients with schizophrenia or alcoholism. Reduced REM sleep latency has been attributed to cholinergic hyperactivity secondary to increased dopaminergic tone in schizophrenia [20]. The most striking finding in the sleep recording was the high amount of deep sleep (SWS: 41.8%, S4 sleep: 26.1%). One of the consistent alterations in normal ageing is a decrease of SWS. The sleep patterns of children are typically characterized by high amounts of SWS, but a quantitative decrease occurs during puberty. As ageing proceeds, a gradual decline in SWS is observed [21]. In normal young adult individual SWS generally constitutes about 13 to 23% and S4 sleep about 10 to 15% of sleep [22], and the amounts of these sleep stages in controls of the present study are in good agreement with this.

**Table 1:** Polysomnography parameters of a homicidal woman with schizophrenia, alcoholismand features of antisocial personality disorder and of three healthy female controls. REM = rapid eye movement sleep, S1–S4 = sleep stages 1–4, SWS = slow wave sleep.

	**patient**	**controls **mean (SD)	**control 1**	**control 2**	**control 3**
time in bed (min)	475.5	562.0 (10.00)	552.0	562.0	572.0
sleep latency (min)	56.0	19.0 (7.26)	18.5	26.5	12.0
sleep period (min)	419.5	543.0 (14.76)	533.5	535.5	560.0
awakenings (n)	14	7.7 (0.58)	7	8	8
wake after sleep onset (min)	56.0	30.3 (2.02)	30.0	28.5	32.5
total sleep time (min)	363.5	512.7 (12.97)	503.5	507.0	527.5
sleep efficiency (%)	86.7	94.4 (0.25)	94.4	94.7	94.2
REM latency (min)	52.5	85.8 (5.01)	81.0	85.5	91.0
S1 (%)	3.3	6.9 (0.90)	6.0	7.8	6.8
S2 (%)	33.2	53.1 (1.66)	51.3	53.3	54.6
S3 (%)	15.7	7.8 (1.41)	7.7	6.5	9.3
S4 (%)	26.1	11.6 (2.07)	14.0	10.7	10.2
SWS (%)	41.8	19.5 (2.25)	21.7	17.2	19.5
REM (%)	21.7	20.6 (1.35)	21.0	21.7	19.1

The finding concerning SWS is completely contrary to previous sleep reports in patients with schizophrenia as well as with alcohol dependence. The patient had abstained from alcohol for several months before the PSG, and this raises the question if the result could be explained with a withdrawal state. However, reduced SWS is also associated also with alcohol withdrawal and the phenomenon has been reported to be extremely long lasting [11]. Even after one or two years of abstinence, the sleep records of alcoholics had partly normalized but the percentage of S4 sleep still remained at lowered levels [23]. Thus, alcohol abstinence cannot explain the finding.

MRI disclosed no post-traumatic signs in the brain substance of the patient. Waking EEG was also within normal limits. Minor head injuries (concussions) are, however, frequent events among people with alcoholism. Kaufman et al. [24] demonstrated a chronic sleep disturbance several years after a minor head injury in a non-selected population. They found lower sleep efficiency, and more awakenings lasting more than three minutes, but no changes in S3 or S4 sleep compared with healthy controls.

The patient had a history of violent acts and her lifestyle was described to be antisocial and unstable and she was diagnosed as having features of antisocial personality disorder in addition to schizophrenia and alcohol dependence. The authors are not aware of previous sleep studies in patients with both schizophrenia and antisocial personality disorder. In a PSG study among habitually violent male offenders with antisocial personality disorder as a primary diagnosis, increased amounts of SWS and S4 sleep were observed as compared with age-matched healthy men [25], which is in agreement with the present case-study. Furthermore, the male offenders with severe conduct disorder preceding ASP had higher amounts of deep sleep than men with only mild or moderate conduct disorder [26]. Adult antisocial personality disorder, childhood conduct disorder [27] and childhood ADHD [28] are the only psychiatric disorders reported to be associated with increased deep sleep. Whether the phenomenon observed in these three disorders reflects specific brain pathology, or a delay in the normal development of sleep patterns in the course of ageing, is still an open question and needs to be clarified.

All patients with schizophrenia should not be considered to be violent, although there are minor subgroups of schizophrenic patients among whom the risk for violence is remarkably high. It has been estimated that this increase in risk could be associated with the paranoid form of schizophrenia, coexisting substance abuse and antisocial behaviour [6]- the disorders, which our patient has. Clozapine, an atypical antipsychotic with significant anti-aggressive effects [29] is widely used in institutions like state mental hospitals where habitually violent patients with schizophrenia are treated. Interestingly, clozapine has been shown to significantly reduce the amount of S4 sleep both in healthy controls [30] and in patients with schizophrenia [31]. Future research is needed to clarify this association and the mechanisms behind this phenomenon.

## Conclusions

Increased deep sleep has been associated with antisocial behaviour with severe aggression. The authors were able to observe this phenomenon in an antisocially behaving, violent female offender with schizophrenia and alcohol dependence, the latter disorders previously reported to be related to low levels of SWS. New studies are, however, needed to confirm and explain this preliminary finding.

## Competing interests

The author(s) declare that they have no competing interests.

## Authors' contributions

This manuscript was prepared by a multidisciplinary team consisting of:

NL, generated the idea for this case report, interviewed the controls, scored the sleep recordings, and prepared the manuscript together with the team.

PT, had a substantial contribution in theoretical background and processing of the present study as expert in sleep research

PT, reviewed all material concerning the patient as expert in forensic psychiatry

ES, participated in processing of the manuscript as expert in forensic psychiatry

HP, participated in the processing of the manuscript as expert in forensic psychiatry and female violence

ME had a central role in planning the study design as well as in formulating the theoretical background of the present study. He also allocated financial resources to this project and helped solving practical problems of the study project

MV, supervised and participated with great impact in all stages of this manuscript

## Pre-publication history

The pre-publication history for this paper can be accessed here:


